# Oral Microbiology – progress, myths and evidence

**DOI:** 10.1080/20002297.2017.1325184

**Published:** 2017-05-25

**Authors:** Mike Curtis

**Affiliations:** ^a^ Institute of Dentistry, Barts and the London, QMUL

## Abstract

This presentation aims to address some of the significant advances in oral microbiology that have taken place since the last EOMW. The use of high throughput, non-cultural approaches to describe the qualitative composition of oral microbial communities in different sites and in different populations, advanced imaging methodologies to define the community architecture of oral biofilms and progress in culturing organisms from the extensive “not yet cultivatable” catalogue are significant highlights. Collectively these approaches are providing unprecedented insights into the nature of the oral microbiome and its putative relationship to oral (and systemic) health and disease. These largely descriptive studies do not in themselves address the aetio-pathological mechanisms at play. However, these too are being progressed through the use of ever more sophisticated *in vitro* and *in vivo* model systems which are enabling a more complete understanding of, at least, the repertoire of host:bacterial interactions in the mouth and their potential relevance to tissue homeostasis versus the development of disease. Whilst there has been undoubted progress, the fundamental requirements for well-designed and appropriately powered clinical studies, experimental rigour in the application and bioinformatic analysis of high throughput molecular assays and the availability of reliable and relevant model systems remain significant barriers.

